# Hospitalization of Pediatric Enteric Fever Cases, Dhaka, Bangladesh, 2017–2019: Incidence and Risk Factors

**DOI:** 10.1093/cid/ciaa1356

**Published:** 2020-12-01

**Authors:** Shampa Saha, K M Ishtiaque Sayeed, Senjuti Saha, Md Shafiqul Islam, Afiour Rahaman, Maksuda Islam, Hafizur Rahman, Raktim Das, Md Mahmudul Hasan, Mohammad Jamal Uddin, Arif Mohammad Tanmoy, A S M Nawshad Uddin Ahmed, Stephen P Luby, Jason R Andrews, Denise O Garrett, Samir K Saha

**Affiliations:** 1 Child Health Research Foundation, Dhaka, Bangladesh; 2 Johns Hopkins Bloomberg School of Public Health, Baltimore, Maryland, USA; 3 Bangladesh Institute of Child Health, Dhaka Shishu Hospital, Sher-E-Bangla Nagar, Dhaka, Bangladesh; 4 Division of Infectious Diseases and Geographic Medicine, Stanford University School of Medicine, Stanford, California, USA; 5 Applied Epidemiology, Sabin Vaccine Institute, Washington, DC, USA

**Keywords:** enteric fever, typhoid, hospitalization, incidence, risk factors

## Abstract

**Background:**

Enteric fever causes substantial morbidity and mortality in low- and middle-income countries. Here, we analyzed Surveillance for Enteric Fever in Asia Project (SEAP) data to estimate the burden of enteric fever hospitalization among children aged <15 years and identify risk factors for hospitalization in Bangladesh.

**Methods:**

SEAP used hospital surveillance paired with a community-based health-care utilization assessment. In SEAP hospital surveillance, blood was obtained for culture from children aged <15 years with ≥3 days of fever. In the hospital catchment area, a health-care utilization survey (HCUS) was conducted to estimate the proportion of febrile children hospitalized at the study hospitals. We analyzed hospital surveillance and HCUS data to estimate the health care–adjusted incidence of enteric fever hospitalization, and conducted univariable and multivariable logistic regressions.

**Results:**

From July 2017 through June 2019, 2243 laboratory-confirmed enteric fever cases were detected in 2 study hospitals; 673 (30%) were hospitalized. The health care–adjusted incidence of enteric fever hospitalization among children <15 years old was 303/100 000 children/year (95% confidence interval [CI], 293–313). *Salmonella* Typhi contributed most to the enteric fever hospitalization incidence (277/100 000 children/year; 95% CI, 267–287). The incidence was highest among children aged 2 to <5 years (552/100 000 children/year; 95% CI, 522–583), followed by those aged <2 years (316/100 000 children/year; 95% CI, 288–344). Factors independently associated with enteric fever hospitalization included fever duration, diarrhea, vomiting, abdominal pain, and leukocytopenia.

**Conclusions:**

We estimated a high burden of hospitalization due to enteric fever among children aged <5 years in Bangladesh. The introduction of a typhoid conjugate vaccine would protect children from typhoid and avert typhoid hospitalizations.

Typhoid and paratyphoid, collectively called enteric fever, are caused by the bacteria *Salmonella enterica* serovars Typhi and Paratyphi A, B, or C. Enteric fever remains a major cause of morbidity and mortality in low- and middle-income countries [1, 2]. In 2017, *S.* Typhi caused approximately 11 million illnesses and 117 000 deaths, whereas *S.* Paratyphi caused over 3 million illnesses and 19 000 deaths globally [3, 4]. The burden of enteric fever was greatest in South Asia and among children aged <15 years [3].

A heat-killed, phenol-preserved, whole-cell typhoid vaccine for the prevention of typhoid fever has been available since 1896. The efficacy of this vaccine was established in 1960 [5–7]. Live attenuated Ty21a and Vi typhoid polysaccharide vaccines are effective against typhoid fever, but these vaccines are not recommended for children aged <2 years and have a short duration of protection [5]. A new-generation typhoid conjugate vaccine (TCV) containing Vi polysaccharide conjugated to a tetanus-toxoid protein carrier showed 55% protective efficacy in a human challenge model [8]. Considering its improved immunologic properties, suitability for use in infants and young children, and expected longer duration of protection, the World Health Organization (WHO) Strategic Advisory Group of Experts recommended the use of TCV over other available vaccines against typhoid in October 2017 [9]. In January 2018, the WHO prequalified the first TCV for use in countries with a high burden of typhoid fever [10]. The Phase III clinical trial of the WHO-prequalified TCV in Nepal from 2017–2019 showed that a single dose of TCV was 82% effective in reducing *S.* Typhi bacteremia in children 9 months to 16 years of age [11].

For the introduction of a new vaccine into a country’s national immunization program, the disease burden is a critical consideration [12]. As the case fatality rate of typhoid fever is less than 1%, hospitalization may serve as a proxy of severe disease [13]. In resource-poor countries, there is a fierce competition for hospital beds. For example, in Bangladesh there are only 3 beds per 10 000 people [14]. The lack of hospital beds often means that patients with serious illnesses—for example, pneumonia, meningitis, birth asphyxia, and preterm birth–related complications—are not admitted [15]. The prevention of hospitalization of enteric fever cases would free up these needed hospital beds, and would ultimately reduce the burden on the health system. Therefore, hospitalization can be a consideration for decision-making for the introduction of TCV in the national immunization program.

In Bangladesh, several studies have documented a high burden of enteric fever at hospitals and in the community [16–20]. However, data on the incidence of enteric fever hospitalization are scarce. Hospitalizations were reported in 2 population-based studies that were conducted in urban slums and were limited by representation of the urban slum population. These studies captured disease at an early stage by active surveillance in the community, hence interrupting the natural course of disease that might have led to hospitalization [19, 20]. The Surveillance for Enteric Fever in Asia Project (SEAP), a multi-country, multi-site prospective study, used a hybrid surveillance approach with facility-based surveillance paired with a community-based health-care utilization survey (HCUS) to assess the burdens of enteric fever in Bangladesh, Pakistan, and Nepal. The community-based HCUS included all socioeconomic levels and did not interrupt the natural course of disease, as it did not involve active, population-based surveillance [21]. Here, we analyzed SEAP data to estimate the burden of enteric fever hospitalization among children aged <15 years in an urban population and to identify risk factors for hospitalization in Bangladesh.

## METHODS

### Hospital Surveillance

#### Study Design, Sites, and Procedures

**Figure 1.**
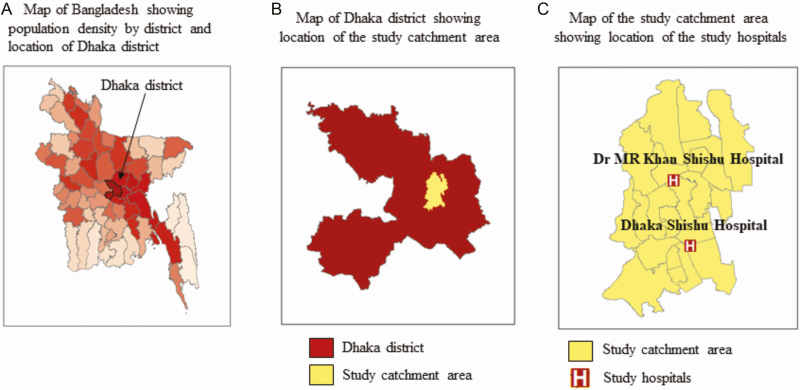
Maps showing population density in Bangladesh by (*A*) districts and location of Dhaka district, (*B*) location of the study catchment area in Dhaka district, and (*C*) location of the study hospitals in the study catchment area.

SEAP surveillance was conducted at 2 pediatric hospitals in Dhaka, Bangladesh: Dhaka Shishu (children) Hospital and Dr MR Khan Shishu Hospital. With 660 beds, Dhaka Shishu Hospital is the largest pediatric hospital in Bangladesh that provides primary to tertiary levels of care to patients aged <18 years. With 250 beds, Dr MR Khan Shishu Hospital is the second largest pediatric hospital in the country and provides patients aged <15 years with primary and secondary levels of care. A catchment area of these 2 hospitals that had been previously identified was used for the HCUS (Figure 1) [21, 22].

We enrolled children from the inpatient department (IPD), outpatient department (OPD), and the hospital laboratory. All children living in the hospital catchment area and presenting at the OPD with ≥3 days fever were eligible to participate in the study. The inpatient eligibility criteria included clinical suspicion of enteric fever by clinicians or a blood culture positive for *S.* Typhi or *S.* Paratyphi A. Eligible cases were enrolled in SEAP IPDs and OPDs if they had a blood culture in the study hospital and gave consent to participate in the study. Additional lab-confirmed cases that were identified at the hospital laboratories and missed at the IPDs and OPDs were enrolled retrospectively in the study as hospital lab–enrolled cases.

Detailed demographic, clinical, and laboratory information of enrolled cases was collected electronically.

Information regarding symptoms of enteric fever—for example, fever start date, diarrhea, vomiting, abdominal pain, cough, constipation, headache, seizure, bloody stool, and difficulty breathing—were collected directly from the patient or caregiver by a study physician at the time of enrollment of the patient into SEAP. Diarrhea was defined as the passage of 3 or more loose or liquid stools per day or more frequent passage than is normal for the individual. Clinical signs were assessed and recorded by the study physician, and laboratory information was collected from hospital medical records.

#### Detection of *S.* Typhi and *S.* Paratyphi in Blood Samples

A sample of 3 milliliters of blood was obtained under aseptic conditions for culture from all children enrolled in this study. Blood culture was performed utilizing the BACTEC (Becton Dickinson and Company) automated culture system. The bottle was incubated at 37^o^ C for a maximum of 5 days. After incubation, positive samples were subcultured on sheep blood, chocolate, and MacConkey agar plates. Standard biochemical tests and agglutination with *Salmonella* serovar–specific antisera (Ramel, Thermo Fisher Scientific) were used to confirm *Salmonella* Typhi/Paratyphi isolates [16].

### Health-care Utilization Survey

The hospital catchment area for the HCUS included 22 out of 90 administrative wards of Dhaka city corporation and covered approximately 51 square kilometers. The catchment area was divided into 2524 clusters by an overlaying geographic information system (GIS grids), and then 100 clusters were randomly chosen for conducting HCUS. In SEAP, 2 HCUSs were conducted; the first during April–July 2017 and the second during September–December 2018, during which a new set of clusters were selected. During both HCUSs, each household included in the selected clusters was approached for interview, with no replacement of households in cases of absence or refusal. We used standardized questionnaires to collect health care–seeking behavior of children aged <18 years who had symptoms consistent with enteric fever—which included fever for >3 days in the last 8 weeks for outpatients and hospitalization due to febrile illness in the last 1 year for inpatient cases—and estimated the proportion of individuals with a febrile illness compatible with enteric fever who sought care at the health facilities participating in SEAP hospital surveillance [21].

### Data Analysis

We analyzed data generated from July 2017 to June 2019 through SEAP hospital surveillance and HCUS. We included children aged <15 years in the analysis. We used medians ± interquartile ranges (IQRs) for summarizing continuous variables and frequencies with percentages for summarizing categorical variables. Comparative statistics included independent samples *t*-tests, chi-square tests, and Fisher’s exact tests, as appropriate. We estimated the number of children aged <15 years living within the catchment area by multiplying the number of children identified in the HCUS by the inverse of the fraction of the area sampled (4.3%).

We estimated the crude incidence rate of enteric fever hospitalization in the hospital catchment area by dividing the number of culture-positive typhoid and paratyphoid cases hospitalized by the population, multiplied by time. We adjusted the crude incidence rate of enteric fever hospitalization for 3 factors: the proportion who sought care at study hospitals (facility coverage), the proportion captured by health facilities (facility capture), and blood-culture sensitivity. Facility coverage was estimated by the household survey and facility capture was estimated at study hospitals. In order to adjust for blood-culture sensitivity, we used the 61% sensitivity of blood culture that was reported by a recent systematic literature review [21]. We estimated the adjusted incidence rate of enteric fever hospitalization by dividing the crude incidence of enteric fever hospitalization by the facility coverage, multiplied by facility capture, multiplied by blood-culture sensitivity.

We calculated 95% confidence interval (CIs) for incidence rates using a normal approximation to Poisson distribution method. For the risk-factor analysis, we included children aged <15 years who were enrolled in SEAP hospital surveillance, regardless of residence. Risk factors associated with hospitalization were analyzed using univariable and multivariable logistic regressions. We conducted 2-tailed statistical tests at an alpha level of 0.05.

### Ethical Consideration

This study was approved by the Ethical Review Committee of the Bangladesh Institute of Child Health, Dhaka. Informed written consent for participation in the study was obtained from the guardian of each child. In addition, assent was obtained from the children aged ≥11 years.

## RESULTS

### Hospital Surveillance

**Figure 2.**
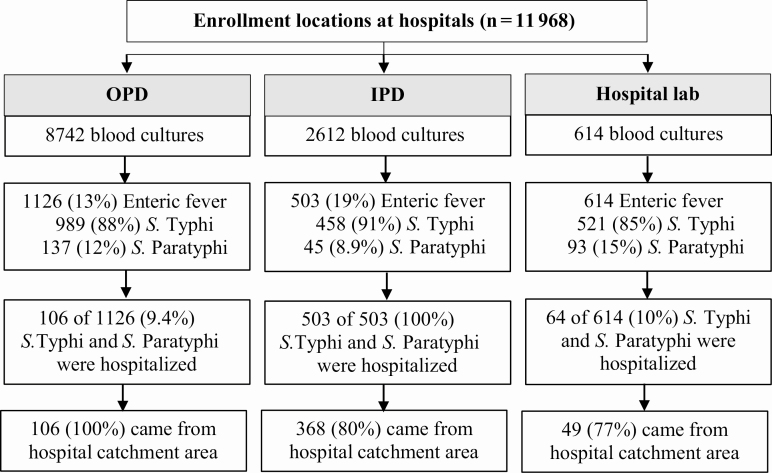
Enteric fever cases aged <15 years enrolled (*n *= 11 968) by recruitment location, with *Salmonella* Typhi and *S.* Paratyphi isolated, and hospitalized at Dhaka Shishu Hospital and Dr MR Khan Shishu Hospital, Dhaka, Bangladesh, July 2017–June 2019. Abbreviations: IPD, inpatient department; OPD, outpatient department; *S*., *Salmonella*.

**Figure 3.**
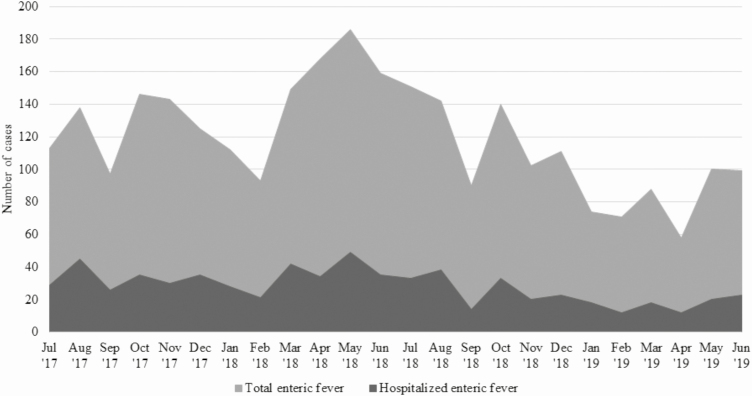
Distribution of total number of enteric fever cases (*n* = 2243) and hospitalizations (*n* = 673) by month at Dhaka Shishu Hospital and Dr MR Khan Shishu Hospital, Dhaka, Bangladesh, July 2017–June 2019.

A total of 11 354 children were enrolled and had a blood culture performed at IPDs and OPDs of Dhaka Shishu Hospital and Dr MR Khan Shishu Hospital (Figure 2). Among these, 1629 lab-confirmed enteric fever cases were detected: 1447 (89%) *S.* Typhi and 182 (11%) *S.* Paratyphi. Additionally, 614 enteric fever cases were identified at the hospital lab who were not enrolled at an IPD or OPD. All 275 *S.* Paratyphi that were isolated during the reporting period were *S.* Paratyphi A. Of the 2243 lab-confirmed typhoid and paratyphoid cases, 673 (30%) were hospitalized, and 91% (n = 612) of the hospitalizations were due to *S.* Typhi. Enteric fever occurred throughout the year, with slight decreases in the numbers of cases and hospitalizations during winter (December–February; Figure 3). The majority (n = 523; 78%) of the hospitalized typhoid and paratyphoid cases came from the hospital catchment area where HCUS had been conducted.

Of 673 hospitalized, lab-confirmed *S.* Typhi and *S.* Paratyphi cases, 361 (54%) were aged <5 years (Table 1). All had fever at the time of hospitalization, with a median duration of 6 days (IQR, 4–8). Additional predominant clinical and laboratory findings at the time of hospitalization included vomiting (262/672, 39%), diarrhea (204/671, 30%), abdominal pain (165/672, 25%), cough (99/672, 15%), hepatomegaly (27/54, 50%), splenomegaly (11/54, 20%), leukocytopenia (25/323, 8%), and thrombocytopenia (25/323, 8%). Nearly all (667/673, 99%) isolates identified from the hospitalized children were ciprofloxacin-resistant, whereas the rate of multidrug resistance, defined as resistance to chloramphenicol, ampicillin, and trimethoprim-sulfamethoxazole, was 17% (115/673). The median duration of stay in the hospital was 7 days (IQR, 5–9). No deaths were reported among the hospitalized cases. Detailed characteristics of hospitalized and nonhospitalized children are shown in Table 1.

**Table 1.** Demographic, Clinical, and Laboratory Characteristics

	Hospitalized		Nonhospitalized			
Characteristics	n	%	n	%	Total	*P* value
Demographic characteristics						
Age						
<2 years	93	14%	176	11%	269	.019^a^
2 to <5 years	268	40%	568	36%	836	
5 to <15 years	312	46%	826	53%	1141	
6 months to <15 years	672	100%	1568	70%	2243	
Median age (IQR) in months	48 (24–84)	…	60 (36–84)	…	60 (36–84)	<.001^a^
Sex						
Male	363	54%	866	55%	1229	.594
Resident of catchment area	523	78%	1438	91%	1961	<.001^a^
Clinical and laboratory findings						
Median duration of fever (IQR) in day at the time of enrollment	7 (5–9)	…	5 (4–8)	…	6 (4–8)	.009^a^
Vomiting	262	39%	274	17%	536	<.001^a^
Diarrhea	204	30%	158	10%	362	<.001^a^
Abdominal pain	165	25%	217	14%	382	<.001^a^
Cough	99	15%	319	20%	418	.006^a^
Constipation	23	3.4%	60	3.8%	83	.615
Headache	18	2.7%	74	4.7%	92	.032^a^
Rash	10	1.5%	5	.3%	15	.006^a^
Jaundice	4	.6%	2	.1%	6	.055
Seizure	3	.4%	1	.1%	4	.055
Bloody stool	2	.3%	0	0%	2	.037^a^
Difficult breathing	2	.3%	4	.3%	6	.376
Hepatomegaly on ultrasonogram	28	52%	5	71%	33	.305
Splenomegaly on ultrasonogram	12	22%	1	14%	13	.645
Leukocytopenia, total WBC < 4000/mm^3^	25	7.7%	10	1.9%	35	<.001^a^
Thrombocytopenia, platelet < 150 000 µl	25	7.7%	13	2.5%	38	<.001^a^
Organism						
*S.* Typhi	612	91%	1356	86%	1968	.003^a^
*S.* Paratyphi	61	9.1%	214	14%	275	
Severity						
Days not able to conduct usual activities due to illness	3 (0–8)	…	3 (0–12)	…	…	.902
Hours of bed rest during worst day of illness	3 (2–6)	…	2 (1–5)	…	…	.003^a^
Drug resistance						
Ampicillin	202	30%	373	24%	575	.002^a^
Chloramphenicol	126	19%	248	16%	374	.088
Cotrimoxazole	118	18%	237	15%	355	.206
Ciprofloxacin	667	99%	1534	97%	2201	.042^a^
Azithromycin	9	1.3%	40	2.5%	49	.072
Cefixime	1	0%	3	0%	4	.827
Ceftazidime	0	0%	0	0%	0	
MDR	115	17%	222	14%	337	.073

Data are of hospitalized (n = 673) and nonhospitalized (n = 1570) S. Typhi and S. Paratyphi cases isolated at Dhaka Shishu Hospital and Dr MR Khan Shishu Hospital, July 2017–June 2019.

Abbreviations: IQR, interquartile range; MDR, multidrug resistance; *S.*, *Salmonella*; WBC, white blood cell.

^a^Statistically significant.

Although young children were more likely to be hospitalized, age was not identified as a risk factor for hospitalization in the multivariable logistic regression. Factors independently associated with hospitalization included fever duration, diarrhea, vomiting, abdominal pain, and leukocytopenia (Table 2). No significant differences in resistance to chloramphenicol, ampicillin, and trimethoprim-sulfamethoxazole were found between hospitalized and nonhospitalized cases.

**Table 2.** Risk Factors for Hospitalization of Children With Laboratory-confirmed Typhoid and Paratyphoid at Dhaka Shishu Hospital and Dr MR Khan Shishu Hospital, Dhaka, Bangladesh, July 2017–June 2019

		Univariable		Multivariable	
Characteristics		Odds Ratio (95% CI)	*P* value	Odds Ratio (95% CI)	*P* value
Age^a^	2 to <5 years	.89 (.67–1.19)	.44	1.02 (.64–1.63)	.938
	5 to <15 years	.71 (.54–.95)	.02	.82 (.52–1.32)	.419
Fever duration		1.01(1.00–1.03)	.025	1.07 (1.03–1.11)	.001^b^
Diarrhea		3.89 (3.09–4.92)	<.001	3.34 (2.26–4.95)	<.001^b^
Vomiting		3.42 (2.53–4.61)	<.001	2.03 (1.44–2.86)	<.001^b^
Abdominal pain		2.03 (1.62–2.54)	<.001	1.62 (1.11–2.37)	.013^b^
Headache		.56 (.33–.94)	.028	.63 (.28–1.38)	.244
Leukocytopenia		4.22 (2–8.91)	<.001	3.34 (1.47–7.59)	.004^b^
Thrombocytopenia		3.22 (1.63–6.41)	.001	2.32 (1.10–4.87)	.027

Abbreviations: CI, confidence interval.

^a^Reference: <2 years.

^b^Statistically significant.

### Community Health-care Utilization

In the hospital catchment area, the estimated number of children aged <15 years in 2017 and 2018 was 1 148 076. During 2 rounds of HCUS, data on hospitalization due to febrile illness in the last 1 year were collected from 36 142 children aged <15 years living in the selected clusters (Table 3). A total of 335 (0.9%) hospitalizations due to febrile illness were identified; 177 (52.8%) were hospitalized at a study hospital.

**Table 3.** Health-care Utilization Survey–Identified Hospitalizations of Children <15 Years Old for Febrile Illness at Dhaka Shishu Hospital and Dr MR Khan Shishu Hospital, Dhaka, Bangladesh, 2017–2018

	HCUS 2017		HCUS 2018		Total	
	*n*	%	*n*	%	*n*	%
<15 year old children interviewed for hospitalization due to febrile illness in last 1 year	20 603	…	15 539	…	36 142	…
Hospitalized anywhere	141	.7%	194	1.2%	335	.9%
Hospitalized at Dhaka Shishu Hospital	22	15.6%	49	25.3%	71	21.2%
Hospitalized at Dr MR Khan Shishu Hospital	53	37.6%	53	27.3%	106	31.6%
Hospitalized at other places	66	46.8%	92	47.4%	158	47.2%

Abbreviation: HCUS, health-care utilization survey.

### Incidence of Hospitalization Due to Enteric Fever

The overall crude incidence of hospitalization due to enteric fever among children aged <15 years in the hospital catchment area in urban Dhaka during 2017–2018 was 46 per 100 000 children per year (95% CI, 42–49; Table 4). The estimated incidence of enteric fever hospitalization after adjustment for facility coverage, facility capture, and blood-culture sensitivity was 303 per 100 000 children per year (95% CI, 293–313). *Salmonella* Typhi was the main contributing factor for hospitalization. There was no difference in the hospitalization rates between males and females. The hospitalization rate was highest among children aged 2 to <5 years, followed by those aged <2 years.

**Table 4.** Crude and Adjusted Incidence Rate of Hospitalization of Laboratory-confirmed *S.* Typhi and *S.* Paratyphi Among Children Aged <15 by Organism, Sex, Age Group and Recruitment Location, at the Catchment Area of Dhaka Shishu Hospital and Dr MR Khan Shishu Hospital, Dhaka, Bangladesh, July 2017–June 2019

Characteristics	Number of hospitalized cases	Population	Crude incidence rate per 100 000/year (95% CI)	Febrile cases hospitalized at study hospitals	Health-care seeking adjusted incidence per 100 000/year (95% CI)	Facility captured febrile cases at study hospitals	Facility capture adjusted incidence per 100 000/year (95% CI)	Blood culture sensitivity	Blood culture sensitivity adjusted incidence per 100 000/year (95% CI)
Overall enteric fever	523	1 148 076	46 (42–49)	53%	86 (81–92)	47%	185 (177–193)	61%	303 (293–313)
Organism									
*S.* Typhi	478	1 148 076	42 (38–45)	53%	79 (74–84)	47%	169 (161–177)	61%	277 (267–287)
*S.* Paratyphi A	45	1 148 076	3.9 (2.8–5.1)	53%	7.4 (5.8–9)	4%	16 (14–18)	61%	26 (23–29)
Sex									
Male	276	584 730	47 (42–53)	54%	88 (80–95)	47%	185 (174–196)	61%	303 (289–317)
Female	247	563 346	44 (38–49)	51%	86 (78–93)	46%	188 (176–199)	61%	308 (293–322)
Age									
<2 years	70	155 915	45 (34–55)	58%	78 (64–92)	40%	193 (171–215)	61%	316 (288–344)
2 to <5 years	209	225 453	93 (80–105)	57%	163 (146–179)	48%	337 (313–361)	61%	552 (522–583)
5 to <15 years	244	766 708	32 (28–36)	46%	69 (63–75)	50%	138 (129–146)	61%	225 (215–236)
6 months to <15 years	522	1 110 388	47 (43–51)	51%	91 (86–97)	47%	195 (187–203)	61%	319 (309–330)
Enrollment location									
OPD	106	1 148 076	9.2 (7.5–11)	53%	17 (15–20)	44%	40 (36–43)	61%	65 (60–70)
IPD	368	1 148 076	32 (29–35)	53%	61 (56–65)	84%	72 (67–77)	61%	118 (112–125)
Hospital lab	49	1 148 076	4.3 (3.1–5.5)	53%	8.1 (6.4–10)	100%	8.1 (6.4–10)	61%	13 (11–15)

Abbreviations: CI, confidence interval; IPD, inpatient department; OPD, outpatient department; *S.*, *Salmonella*.

## Discussion

Our data showed, over the 2-year study period, that 30% of the lab-confirmed pediatric enteric fever cases visiting the study hospitals required hospitalization. The incidence of enteric fever hospitalization was 303 per 100 000 children per year; *S.* Typhi was the main contributing factor for hospitalization. The hospitalization rate was highest among children aged 2 to <5 years, followed by those aged <2 years. The duration of fever and the presence of diarrhea, vomiting, abdominal pain, and leukocytopenia were identified as risk factors for enteric fever hospitalization.

Similar to our findings of 30% hospitalization, a study conducted from 2004 through 2016 in a similar setting reported that 32% of the typhoid cases and 21% of the paratyphoid pediatric cases required hospitalization [16]. Previous data showed that 3.6% of all admissions at large pediatric hospitals in Bangladesh were either laboratory-confirmed or clinically diagnosed enteric fever cases [23]. This could be because Bangladesh has a high burden of enteric fever, with incidence rates ranging from 2.7–18.7 per 1000 children [19, 20, 24].

In concordance with previous studies evaluating the clinical profile of enteric fever, we found that the predominant signs, symptoms, and laboratory findings of hospitalized enteric fever cases include fever, vomiting, diarrhea, abdominal pain, cough, hepatomegaly, and splenomegaly [23, 25–27]. But in contrast to the systematic review conducted by Azmatullah et al. [27] and a study conducted in Fiji [26], we observed lower proportions of children with signs and symptoms of complications, including gastrointestinal bleeding (6–8% in prior studies vs 0.3% in our study) and jaundice (2.8% in prior studies vs 0.6% in our study), and no mortality (1–6% in prior studies vs 0% in our study) due to enteric fever among children, which is possibly due to the widespread over-the-counter availability of antibiotics in Bangladesh. Relatively less severe disease in our population could also possibly be explained by a hypothesis that frequent subclinical exposure to *S.* Typhi and *S.* Paratyphi from contaminated water and food may provide a level of immunity against the disease to the people living in the endemic area. However, our data demonstrated that only fever duration, diarrhea, vomiting, abdominal pain, and leukocytopenia were associated with enteric fever hospitalization among children aged <15 years. Although there are reports on coinfections with dengue, malaria, and so forth, data on the role of coinfection with other pathogens on hospitalization of enteric fever cases is scarce [28, 29]. In the blood cultures of the enteric fever cases enrolled in SEAP, we did not find any other bacterial pathogens. However, based on the clinical criteria and advice of clinicians, only 74 cases were tested for dengue. Of them, 2 (2.7%) were positive for either an NS1 antigen test or anti-dengue immunoglobin M. As the number of dengue-positive cases was too small, we did not include coinfection with dengue in the risk factor analysis.

We reported that the adjusted incidence of hospitalization due to typhoid and paratyphoid was 303 per 100 000 children per year in children aged <15 years. This rate is higher than the rates observed by previous studies conducted in Bangladesh, Kenya, and Spain [19, 20, 25, 30]. In Bangladesh, Brooks et al. [20] gathered 12 407 person-years worth of data for 10 months, but found no hospitalizations, complications, or deaths among blood culture–confirmed typhoid fever patients. Naheed et al. [19] observed only 4 hospitalizations among blood culture–confirmed typhoid fever patients over 1 year and 19 710 person-years worth of data. This translates to 20 enteric fever hospitalizations per 100 000 population per year. These studies utilized active, population-based surveillance that captured cases at an early stage of the disease and, thus, prevented hospitalization by treating them early. Our study utilized a hybrid method that did not interfere in the natural course of the disease and included a combination of passive surveillance at the study hospitals and HCUS in the hospital catchment area for adjustment in the care seeking seen at the hospitals.

Our study demonstrated the substantial burden of enteric fever in children aged <15 years, with the greatest burden in children aged <5 years, which is consistent with other studies [19, 20, 24, 30]. We estimated 277 hospitalizations due to *S.* Typhi per 100 000 children per year, and a median of 7 days of hospital stay. This translates to 3180 hospitalizations due to typhoid, with an estimated 22 261 hospital bed-days in a year in the hospital catchment area. This has important policy implications, as a TCV with 80% efficacy and 90% vaccination coverage would be able to avert 16 028 hospital bed-days per year in the hospital catchment area. The resulting availability of hospital beds at secondary- and tertiary-level hospitals would mean children with other serious illnesses, at risk of disability and mortality, would have the opportunity to receive treatment at the hospital [15]. Thus, TCV would reduce the burden on the overall health system.

Our study included all hospital departments: OPDs, IPDs, and hospital laboratories. Although most of the cases contributing to the numerator came from the IPDs, the inclusion of OPDs and hospital labs into the study increased the capture of cases by 40%. This finding illustrates the importance of a comprehensive surveillance system for the estimation of the disease burden [31].

This study had some limitations. First, this surveillance was conducted in a city where we know the water is contaminated with *S.* Typhi [32]. Therefore, the catchment area of the study hospitals may be a high-incidence area for enteric fever. The burden of the disease in other communities is likely to be different. Therefore, we would not extrapolate this incidence rate to a national estimate. However, our study includes a representative urban population of all socioeconomic levels. Second, we used a 61% sensitivity rate to adjust the incidence of enteric fever hospitalization for all subgroups, as information on sensitivity rates by subgroups was not available. Sensitivity rates may vary by subgroups, and thus affect our estimates for different subgroups.

## Conclusions

We estimated that 303 per 100 000 children aged <15 years with enteric fever require hospitalization per year. Children aged <5 years are affected most. The introduction of TCV in the national immunization program would protect children from typhoid and avert related admissions at pediatric hospitals, thus allowing children with other severe diseases to be treated. Thus, TCV would not just prevent morbidity and mortality due to typhoid fever, it would also save more lives and prevent disability from other severe diseases, and therefore reduce the burden on the health system and improve child health overall.

## Notes

***Acknowledgments.*** The authors thank all physicians, laboratory personnel, and research assistants working at Dhaka Shishu Hospital and Dr MR Khan Shishu Hospital, especially Dr Dipalok Mukharjee, Dr Shamsun Nahar, Dr Shammin Azmery, Dr Humaira Susmita, Dr Shumaiya Ferdaus, and Mr Anik Sarker, who played active roles in implementation of the Surveillance for Enteric Fever in Asia Project hospital surveillance; Ms Salma Akhter for coordination of the health-care utilization survey (HCUS); all research assistants who collected quality HCUS data; Ms Ruma Datta for administration support; and Mr. Md. Shariful Islam and Mr Argho Sarker for data management support.

***Financial support.*** This work was supported by the Bill and Melinda Gates Foundation (Investment ID OPP1113007).

***Supplement sponsorship.*** This supplement is sponsored by the Sabin Vaccine Institute and made possible by a grant from the Bill & Melinda Gates Foundation.

***Potential conflicts of interest.*** The authors: No reported conflicts of interest. All authors have submitted the ICMJE Form for Disclosure of Potential Conflicts of Interest.
